# Nonsurgical Providers Provide the Majority of Postoperative Opioid Prescriptions After Hand Surgery

**DOI:** 10.7759/cureus.15564

**Published:** 2021-06-10

**Authors:** Madeline Tadley, Clay B Townsend, Shivangi Bhatt, Monica Morgenstern, Kevin F Lutsky, Pedro K Beredjiklian

**Affiliations:** 1 Orthopaedic Surgery, Rothman Orthopaedic Institute, Philadelphia, USA; 2 Medicine, Drexel University College of Medicine, Philadelphia, USA; 3 Orthopaedic Surgery, University of Vermont, Burlington, USA; 4 Division of Hand Surgery, Rothman Orthopaedic Institute, Philadelphia, USA

**Keywords:** hand surgery, opioid refills, opioid prescription, orthopaedic surgery, postoperative pain

## Abstract

Introduction

The increased use of Prescription Drug Monitoring Program (PDMP) websites has helped physicians to limit overlapping controlled substance prescriptions and help prevent opioid abuse. Many studies have investigated risk factors for prolonged opioid use after orthopedic surgery, but few studies have investigated who is prescribing opioids to postoperative patients. The purpose of this study is to investigate the types of medical providers prescribing opioids to hand surgery patients postoperatively.

Methods

Institutional Review Board approval was obtained prior to initiation of this study. An institutional database search was performed to identify all patients ≥18 years old that underwent a single hand surgery at our institution during a specified time period. Patients with more than one surgical procedure during this time were excluded to prevent potential crossover with opioid prescriptions for different surgical procedures. A search of the state PDMP website was performed to identify opioid prescriptions filled by hand surgery patients from six months preoperatively to 12 months postoperatively. Opioid prescribers were classified into several groups: 1) the patient’s operating surgeon, 2) other orthopedic surgery providers, 3) general medicine providers (internal medicine, primary care, family medicine, and adult health providers), and 4) all other medical providers.

Results

Three hundred twenty-seven patients could be identified in the PDMP database who received an opioid prescription on the day of surgery. Of these, 108 (33.0%) filled a total of 341 additional opioid prescriptions postoperatively. Non-orthopedic providers prescribed 81.5% of all opioid prescriptions within 12 months postoperatively, with the patient’s operating surgeon prescribing only 10% of all prescriptions. General medicine providers were the highest prescriber group at 28.7% of total postoperative opioid prescriptions. From six to 12 months postoperatively, the patient’s operating surgeon prescribed only 4.9% of total opioid prescriptions filled. The patient’s operating surgeon prescribed significantly smaller average opioid prescriptions in total morphine milligram equivalents compared to all other provider groups.

Conclusions

Surgeons should be aware that their surgical patients may be receiving opioid prescriptions from a wide variety of medical providers postoperatively, and that these other providers may be prescribing larger prescriptions. The findings of this study emphasize the importance of collaboration across medical specialties to mitigate the risks of prolonged opioid use after hand surgery.

## Introduction

The drug overdose death rate in the United States has more than tripled from 1999 to 2018 [1], with about 70% of drug overdose deaths in 2018 involving an opioid [2]. This public health crisis has prompted increased awareness by providers to try to limit opioid prescriptions to their patients. The use of Prescription Drug Monitoring Program (PDMP) websites allows physicians to better monitor controlled substance prescriptions and to help avoid patients obtaining opioids from multiple providers simultaneously. Increased PDMP utilization could have possibly contributed in part to the 13.5% decrease in the prescription opioid-involved death rate from 2017 to 2018 [2].

Recent studies have investigated risk factors for prolonged postoperative opioid use after orthopedic surgery [3,4]. However, the actual sources of postoperative opioid prescriptions are rarely reported. Studies on opioid use in orthopedic surgery patients have found that a substantial number of postoperative opioid prescriptions are written by providers other than the orthopedic surgeons who had performed the surgical procedure [5-7]. 

There are currently limited data to help guide our knowledge of where patients receive postoperative opioid prescriptions after hand surgery. The purpose of this study is to investigate the types of providers prescribing opioids to hand surgery patients in the postoperative period. We hypothesized that the patient’s operating surgeon would be writing the majority of prescriptions in the immediate postoperative period, but that patients would be receiving more opioid prescriptions from non-orthopedic providers as time increased from surgery.

## Materials and methods

Institutional Review Board approval was obtained prior to initiation of this study, with a waiver of informed consent per institutional protocol. An institutional database search was performed to identify all patients ≥18 years old who underwent a single hand surgery at our institution from July 31, 2018 to March 15, 2019. Patients with more than one surgical procedure during this time were excluded to prevent potential crossover with opioid prescriptions for different surgical procedures. Only patients who received an opioid prescription on the day of surgery were included in the analysis.

The Pennsylvania (PA) PDMP website was queried using patient name and date of birth for all prescriptions filled from six months preoperatively to 12 months postoperatively for all patients. The PA PDMP website includes prescription information from the following states: PA, Arkansas, Connecticut, Delaware, Florida, Louisiana, Maine, Maryland, Massachusetts, Military Health System, Minnesota, New York, North Carolina, Ohio, Oklahoma, Rhode Island, South Carolina, Texas, Virginia, and West Virginia. Information recorded from the PDMP website included date the prescription was written, date the prescription was filled, medication name, morphine milligram equivalents (MMEs) of the prescription, name of the prescriber, and prescriber office address. An internet search was then performed to identify the medical specialty of all providers who wrote an opioid prescription for a hand surgery patient within 12 months postoperatively. The patient’s initial postoperative opioid prescription (written by the operating surgeon) was not included in this prescription count. The providers who prescribed the postoperative opioids were divided into several groups: 1) the patient’s operating surgeon, 2) other orthopedic surgery providers, 3) general medicine providers (internal medicine, primary care, family medicine, and adult health providers), and 4) all other medical providers.

The number of opioid prescriptions written by different medical providers was summarized with counts and percentages and was compared with chi-square testing. Prescription size in total MMEs was summarized with averages and compared with independent t-test. Statistical significance was empirically established at a p-value <0.05. 

## Results

During the study period, 457 patients underwent a single hand surgical procedure. Of these, 327 patients could be identified in the PA PDMP system and also received an opioid prescription on the day of surgery. Of these 327 patients, 108 patients (33.0%) received at least one additional opioid prescription within 12 months postoperatively. 

Of these 108 patients, 46 (42.6%) had been given a total of 125 opioid prescriptions in the six months prior to their surgery. Nine of these prescriptions (7.2%) were prescribed by the surgeon who eventually performed the patient's surgery, nine (7.2%) were prescribed by another orthopedic surgery provider, 40 (32%) were prescribed by a general medicine provider, and 67 (53.6%) were prescribed by other medical providers (Table 1). Of these 46 patients with preoperative opioid exposure who filled additional prescriptions, 20 (43.5%) received an opioid prescription postoperatively from a new medical provider from whom they had not received opioids from preoperatively.

**Table 1:** Opioid prescribers of all opioid prescriptions filled within six months preoperatively MME = morphine milligram equivalent; PM&R = physical medicine and rehabilitation.

Provider Type	N (%)	Average MMEs per Rx
General Medicine	40 (32.0)	374.9
PM&R	21 (16.8)	469.6
Emergency Medicine	10 (8.0)	98.7
Operating Surgeon	9 (7.2)	179.2
Other Orthopedic Surgery Provider	9 (7.2)	235.0
Rheumatology	9 (7.2)	783.3
Dentist	7 (5.6)	60.2
Anesthesiology	6 (4.8)	1800.0
Sleep Medicine	6 (4.8)	450.0
General Surgery	3 (2.4)	175.0
Pain Management	2 (1.6)	562.5
Pediatrics	2 (1.6)	300.0
Acute Care	1 (0.8)	400.0

The 108 patients who received at least one additional opioid prescription postoperatively filled a total of 341 additional opioid prescriptions. The distribution of postoperative opioid prescribers by medical specialty is listed in Table 2. Of the 341 total postoperative opioid prescriptions filled, 34 (10.0%) were prescribed by the patient’s operating surgeon, 29 (8.5%) were prescribed by another orthopedic surgery provider, 98 (28.7%) were prescribed by a general medicine provider, and 180 (52.8%) were prescribed by other medical providers (Table 2). There were 127 distinct providers who prescribed opioids to the surgical patients during the 12-month postoperative period. The patient’s operating surgeon prescribed significantly smaller opioid prescriptions during the postoperative period compared to other orthopedic surgery providers (average 145.2 MMEs vs 237.1 MMEs; p < 0.01), compared to general medicine providers (average 145.2 MMEs vs 341.4 MMEs; p < 0.01), and compared to all other medical providers (average 145.2 MMEs vs 475.6 MMEs; p < 0.01) (Table 2). 

**Table 2:** Opioid prescribers of all opioid prescriptions filled within 12 months postoperatively MME = morphine milligram equivalent; PM&R = physical medicine and rehabilitation; ENT = ear, nose, and throat; OBGYN = obstetrics and gynecology.

Provider Type	N (%)	Average MMEs per Rx
General Medicine	98 (28.7)	341.4
PM&R	52 (15.2)	493.9
Operating Surgeon	34 (10.0)	145.2
Other Orthopedic Surgery Provider	29 (8.5)	237.1
Anesthesiology	21 (6.2)	976.4
Dentist	18 (5.3)	79.5
Rheumatology	15 (4.4)	600.0
General Surgery	12 (3.5)	134.4
Sleep Medicine	12 (3.5)	450.0
Pain Management	10 (2.9)	1635.0
Emergency Medicine	10 (2.9)	431.6
Oncology	7 (2.1)	435.7
Urology	6 (1.8)	1277.5
Acute Care	4 (1.2)	350.0
ENT	4 (1.2)	92.5
Podiatry	2 (0.6)	112.5
Hospice	2 (0.6)	168.8
Pulmonology	1 (0.3)	150.0
Neurology	1 (0.3)	1260.0
OBGYN	1 (0.3)	80.0
Pediatrics	1 (0.3)	300.0
Geriatric Medicine	1 (0.3)	78.8

**Figure 1:**
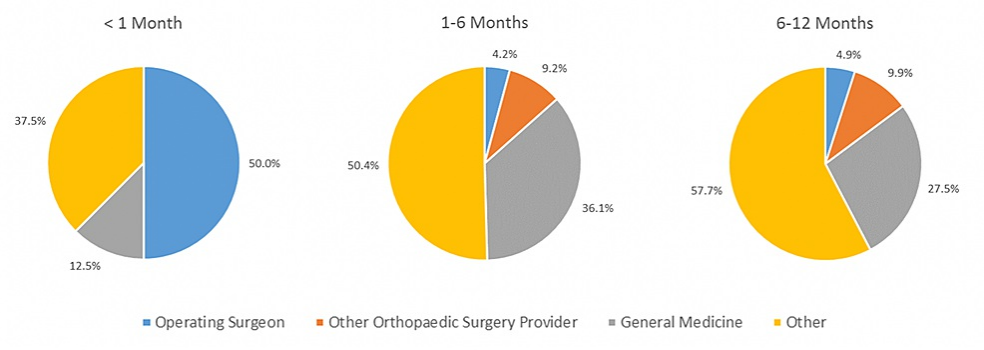
Opioid prescribers based on time from surgery date Other = all other medical specialties.

Prescriptions were further analyzed based on the time from surgery: <1 month, one to six months, and six to 12 months postoperatively (Table 3, Figure 1). The progressive decrease in percentage of prescriptions written by the patient’s operating surgeon over time was statistically significant (20/40 prescriptions at <1 month postoperatively, 5/119 prescriptions from one to six months, 9/182 prescriptions from six to 12 months; p < 0.01).

**Table 3:** Opioid prescribers based on time from surgery date PM&R = physical medicine and rehabilitation; ENT = ear, nose, and throat; OBGYN = obstetrics and gynecology.

Provider Type	<1 Month	1-6 Months	6-12 Months
General Medicine	5 (12.5%)	43 (36.1%)	50 (27.5%)
PM&R	4 (10.0%)	20 (16.8%)	28 (15.4%)
Operating Surgeon	20 (50.0%)	5 (4.2%)	9 (4.9%)
Other Orthopedic Surgery Provider	0 (0.0%)	11 (9.2%)	18 (9.9%)
Anesthesiology	1 (2.5%)	9 (7.6%)	11 (6.0%)
Dentist	2 (5.0%)	5 (4.2%)	11 (6.0%)
Rheumatology	2 (5.0%)	7 (5.9%)	6 (3.3%)
General Surgery	1 (2.5%)	1 (0.8%)	10 (5.5%)
Sleep Medicine	1 (2.5%)	5 (4.2%)	6 (3.3%)
Pain Management	1 (2.5%)	3 (2.5%)	6 (3.3%)
Emergency Medicine	0 (0.0%)	5 (4.2%)	5 (2.7%)
Oncology	1 (2.5%)	3 (2.5%)	3 (1.6%)
Urology	0 (0.0%)	0 (0.0%)	6 (3.3%)
Acute Care	0 (0.0%)	0 (0.0%)	4 (2.2%)
ENT	0 (0.0%)	1 (0.8%)	3 (1.6%)
Podiatry	0 (0.0%)	0 (0.0%)	2 (1.1%)
Hospice	0 (0.0%)	0 (0.0%)	2 (1.1%)
Pulmonology	0 (0.0%)	0 (0.0%)	1 (0.5%)
Neurology	1 (2.5%)	0 (0.0%)	0 (0.0%)
OBGYN	0 (0.0%)	0 (0.0%)	1 (0.5%)
Pediatrics	1 (2.5%)	0 (0.0%)	0 (0.0%)
Geriatric Medicine	0 (0.0%)	1 (0.8%)	0 (0.0%)
Total	40	119	182
Prescriptions per One-Month Period	40	23.8	30.3

## Discussion

Prolonged opioid use after hand surgery is a challenge for both patients and surgeons [8]. Adequate pain management is essential in the postoperative period; however, prolonged unnecessary use of opioids can lead to abuse [9-11]. We sought to identify the types of providers from whom hand surgery patients received opioid prescriptions postoperatively. We found that orthopedic surgery providers wrote less than one-fifth of opioid prescriptions filled by hand surgery patients within 12 months postoperatively, and that the patient’s surgeon wrote only one-tenth of overall prescriptions filled. Based on these findings, while orthopedic surgeons can have an influence on patients’ postoperative opioid usage, the operating surgeon appears to play a relatively small part in providing opioids to their surgical patients.

General medicine providers represented the highest prescriber group, prescribing 28.7% of total prescriptions in this study. This finding is consistent with the reports of opioid prescription patterns among total joint arthroplasty patients. Zarling et al. reported that from three months preoperatively to 12 months postoperatively, 49% of opioid prescriptions were written by primary care physicians [7]. Similar to our findings, they also found that less than one-quarter (23%) of opioid prescriptions during this perioperative time period were written by orthopedic surgeons. This trend appears consistent even in nonsurgical patients with orthopedic complaints. Johnson et al. found that in patients treated nonoperatively for osteoarthritis, 92% of opioid prescriptions were written by non-orthopedic providers [12]. 

Our study revealed that the percentage of postoperative opioid prescriptions written by orthopedic surgery providers decreased significantly from <1 month postoperatively to six to 12 months postoperatively (Figure 1). Surprisingly, only 4.9% of the opioid prescriptions filled from six to 12 months postoperatively were written by the patient’s original surgeon. In total knee arthroplasty patients, Namba et al. found that orthopedic surgeons wrote 47% of opioid prescriptions from zero to three months postoperatively, which decreased to 14% from nine to 12 months postoperatively [5]. Similarly, in total hip arthroplasty patients, they found that orthopedic surgeons wrote 40% of opioid prescriptions from zero to three months postoperatively, which decreased to 14% from nine to 12 months postoperatively. Utilizing a state PDMP database, Rodriguez-Buitrago et al. found that 39% of opioid prescriptions filled by pilon fracture repair patients within six months postoperatively were written by non-orthopedic providers [6]. These findings reflect the episodic role the orthopedic surgeon may have in the long-term health care of their postoperative patients. 

It is possible that patients who received opioid prescriptions in addition to the index prescription did so to treat postoperative pain, a comorbidity that developed postoperatively, or a medical condition that was present prior to surgery. Our data do not allow for an assessment of why or for what condition the patients were prescribed their opioid. One may assume that in the immediate postoperative period, patients will seek additional opioid prescriptions from their surgeon. This is consistent with our finding that the highest prescribers in the acute <1 month postoperative period were orthopedic surgery providers. As time from surgery increases, the surgeon may not feel that continued opioid use is warranted or safe. Although this is not the case at our institution, many institutions have adopted policies that limit the amount of time a patient may receive narcotic prescriptions postoperatively. Standardized opioid policies are intended to not only minimize opioid use but also to set patient expectations. Despite these practices or policies, patients may still feel the need for opioid medications and seek prescriptions from other providers. General medicine providers, who typically have a more sustained relationship with patients, may feel more compelled to provide opioid prescriptions. 

We found that the highest number of filled opioid prescriptions averaged per one-month time period was in the acute <1 month period (Table 3). This finding is realistic given the patients’ recent surgery and the potential requirement for additional pain control. It was concerning that the average number of opioid prescriptions filled per one-month time period had actually increased from 1-6 months to 6-12 months postoperatively. Deyo et al. investigated opioid prescriptions and cumulative MMEs dispensed during the first 30 days following opioid use initiation in any medical patient and discovered that even one additional prescription after the index prescription was associated with 2.25 greater odds of long-term opioid use [13]. Orthopedic surgeons should be aware that as the index opioid prescriber they can help mitigate the risks of opioid use by prescribing responsibly and inquiring about opioid use at postoperative follow-up visits. 

Several studies have found that preoperative exposure to controlled substances, including opioids, are predictive of prolonged postoperative opioid use [4,14,15]. We found that of the 108 patients who had received an additional opioid prescription postoperatively, 46 (42.6%) had received at least one opioid prescription within six months preoperatively. It is possible that preoperatively exposed patients had been receiving opioids for chronic conditions prior to surgery, and these patients could have returned to their long-term providers for refills instead of returning to their surgeon. Situations such as this highlight the advantages of PDMP databases in determining if, and from whom, patients are receiving controlled substance prescriptions. However, even when utilizing PDMP databases, it is still possible that patients receive opioid prescriptions from multiple providers without ensuring that the prescriptions are for different or new problems. Our study found that of the 46 patients who had preoperative opioid exposure and filled additional prescriptions postoperatively, 20 (43.5%) received an opioid prescription from a de novo opioid prescriber postoperatively. In an analysis of the Michigan PDMP database, Zarling et al. discovered that one-third of total joint arthroplasty patients had three or more opioid prescribers from three months preoperatively to 12 months postoperatively [7].

Of the 327 total patients in this study who received an opioid prescription on their surgery date, 62 (19%) filled at least one additional opioid prescription postoperatively without having any exposure to opioids within six months preoperatively. This finding illustrates that the risk for additional filled opioid prescriptions is present even for patients without preoperative exposure. Another study similarly found that 13% of preoperatively opioid-naïve hand surgery patients continued to fill opioid prescriptions from three to six months postoperatively [10]. The PDMP does not provide the medical indications for filled opioid prescriptions. It is possible that some of these patients may have undergone additional procedures at another institution or sustained traumatic injuries within the first year after hand surgery, and thus required opioid prescriptions for pain control. 

Limitations of this study include its retrospective design. We evaluated the number of opioid prescriptions filled by the patients, and this does not necessarily correlate with actual patient opioid consumption. We were also unable to investigate the indications for additional postoperative opioid prescriptions, and it is possible that the patients had subsequent injuries or underwent surgery at other institutions. All included patients were treated at the same institution in the same state, and it is possible that opioid prescribing patterns may vary in different geographic areas. We were not able to control for surgery type, and our data may be reflective of a selection bias in that patients prescribed opioids may have been likely to have a more significant surgery or injury than patients who were not given an opioid on the day of surgery. 

## Conclusions

While significant effort has been made to identify patient risk factors for prolonged postoperative opioid use, much less attention has been directed toward the opioid prescribing patterns of the providers caring for postoperative patients. Surgeons should be aware that patients may be prescribed opioids from a wide variety of medical providers, and that this additional opioid use may affect postoperative opioid use. The findings of this study emphasize the importance of collaboration across medical specialties to mitigate the risks of prolonged opioid use after hand surgery.
